# Effects of photodynamic hyperthermal therapy with indocyanine green on tumor growth in a colon 26 tumor-bearing mouse model

**DOI:** 10.3892/ol.2014.1865

**Published:** 2014-02-10

**Authors:** MASAKI ONOYAMA, KAZUO AZUMA, TAKESHI TSUKA, TOMOHIRO IMAGAWA, TOMOHIRO OSAKI, SABURO MINAMI, NOBUHIKO OGAWA, YOSHIHARU OKAMOTO

**Affiliations:** 1 The United Graduate School of Veterinary Science, Yamaguchi University, Yamaguchi, Yamaguchi 753-8515, Japan; 2 Department of Veterinary Surgery, Faculty of Agriculture, Tottori University, Tottori-shi, Tottori 680-8553, Japan; 3 Department of Veterinary Diagnostic Imaging, Faculty of Agriculture, Tottori University, Tottori-shi, Tottori 680-8553, Japan; 4 Tokyo Iken Co., Ltd., Inashiro-shi, Tokyo 206-0802, Japan; 5 Department of Veterinary Neurology and Oncology, Faculty of Agriculture, Tottori University, Tottori-shi, Tottori 680-8553, Japan

**Keywords:** photodynamic hyperthermal therapy, tumor, indocyanine green, colon 26

## Abstract

The present study used indocyanine green (ICG) and a broadband light source apparatus [photodynamic hyperthermal therapy (PHT) group] in order to treat a colon 26 tumor-bearing mouse model. The other groups were administered either ICG alone (ICG group), light alone (light group) or no treatment (control group). Following the treatment, tumor growth was measured. Nine days after the treatment, the tumors were resected and histological and immunohistological examinations were performed. In the PHT group, the growth rates of the tumor tissues were significantly decreased compared with those observed in the other groups (P<0.05). The proportion of necrotic areas in the PHT and light groups were increased significantly compared with those observed in the ICG and control groups. However, there were no significant differences between the PHT and light groups. The proportion of Ki-67 in the PHT and light groups was less than that observed in the ICG and control groups. The number of terminal deoxynucleotidyltransferase-mediated dUTP nick end labeling-positive cells in the PHT group was significantly increased compared with that observed in the other groups. These data indicate that PHT is effective *in vivo* and *in vitro*.

## Introduction

The life span of animals has grown longer due to vaccinations for various infectious diseases, improvements in food and environment and the development of veterinary medicine. As a result, the incidence of various illnesses that are associated with aging have been increasing in pet populations. In particular, cancer is a significant problem. As in human medicine, there are three major treatments for cancer in veterinary medicine, surgery, chemotherapy and radiation. However, it is difficult to treat all cases with these therapies. Therefore, it is necessary to develop new treatments.

Indocyanine green (ICG) generates heat in response to light near a wavelength of 800 nm (hyperthermia) (1–4). Furthermore, ICG generates active oxygen in response to light at 600–800 nm (photodynamic effect) (5–8). The principle advantage of ICG is low toxicity (9). ICG has become widely used during sentinel biopsies (10 12). Based on this, we developed a new cancer therapy, called photodynamic hyperthermal therapy (PHT), using ICG and a broadband light source apparatus. We previously reported that PHT induced morphological cell death and inhibited the proliferation of the murine B16F10 melanoma cell line (13). Furthermore, it has been demonstrated that PHT induces apoptosis and cell cycle arrest *in vitro* (14). However, there are no *in vivo* experimental data with regard to PHT-related tumor growth and histological changes.

The present study aimed to investigate the effects of PHT on tumor growth and histological changes using colon 26 tumor-bearing mice *in vivo*.

## Materials and methods

### Preparation of the tumor-bearing mouse model

A total of 23 female five-week-old BALB/c mice were purchased from CLEA Japan, Inc. (Osaka, Japan). The animals were maintained under conventional conditions. The use of these animals and the procedures they underwent were approved by the Animal Research Committee of Tottori University. Colon 26 tissue, which is of murine colon cancer origin, was transplanted subcutaneously into the dorsal regions of the mice.

The mice were bred for nine days with free access to food and water, following which, the experiments were performed. The mice whose tumors grew to 5 mm in size were used in this study.

### Study design

The mice (n=23) were divided into four groups that were subjected to light + ICG (PHT group; n=8), ICG alone (ICG group; n=5), light alone (light group; n=5) or were untreated (control group; n=5).

All the treatments were performed at day 0 under general anesthesia with inhalation of 5% isoflurane. In the PHT group, 25 mg ICG (Diagnogreen; Daiichi Sankyo, Tokyo, Japan) was dissolved in 10 ml saline and adjusted to pH 5.0. A total of 0.5 ml ICG solution was injected into the tumor tissue, following which, irradiation was performed using a near-infrared light source (Super Lizer™, Hyper 5000; maximum output, 5.0 W; 600–1600 nm of output wavelength bands; Tokyo Iken Co., Ltd., Tokyo, Japan). Irradiation was performed for 10 min using 20% output so that the distance from the tumor to the light source was 3–5 cm. The temperature of the tumor tissue and on the tumor surface during irradiation was measured using a digital temperature indicator (Anritsu Meter Co., Ltd., Tokyo, Japan) and maintained in a range of 42.5–45.0°C in the tumor and <45.0°C on the tumor surface. In the ICG group, the ICG solution was injected without the administration of irradiation. In the light group, irradiation was performed under similar conditions to those that were used in the PHT group.

Following nine days of treatment (day 9), all the mice were sacrificed by inhalation of 5% isoflurane followed by cervical dislocation. On days 0 and 9, the volume of tumor tissue was calculated by measuring the mediastinum and the transverse length, and the depth of the tumor. Based on volumes of the tumor on days 0 and 9, the tumor growth rate (mm^3^/day) was calculated as follows: (tumor volume on day 9 - tumor volume on day 0)/9. The tumor tissue was removed and fixed in 10% buffered formalin.

### Histological examination

The fixed samples were embedded in paraffin and sectioned in a routine manner. The sections were stained with hematoxylin and eosin (HE staining) and examined immunohistologically for Ki-67 and terminal deoxynucleotidyltransferase-mediated dUTP nick end labeling (TUNEL) staining.

For the Ki-67 staining, 3-μm tissue sections were placed on glass slides and deparaffinized, then washed with ethanol and water and soaked in phosphate-buffered saline (PBS). The sections were autoclaved using 0.01 M citrate buffer (pH 6.0) for 15 min at 121°C, washed with PBS and incubated with rabbit polyclonal anti-Ki-67 antibodies (1:50; code no. E0468; Dako, Glostrup, Denmark) for 30 min at room temperature. Subsequent to being washed with PBS, the sections were incubated with rat anti-immunoglobulin G antibodies (1:100; sc-372; Vector Laboratories, Inc., Burlingame, CA, USA) for 30 min at room temperature. The slides were washed with PBS and avidin/biotin complex methods were performed (PK-4000; Vector Laboratories, Inc.) for 30 min. The tissue sections were counterstained with histogreen and then stained with nuclear fast red.

For the TUNEL staining, 3-μm tissue sections were placed on glass slides and deparaffinized, then washed with ethanol and water and soaked in diluted water. The TUNEL staining was performed using an *In situ* Apoptosis Detection kit (Takara Bio, Inc., Shiga, Japan) according to the manufacturer’s instructions. The tissue sections were counterstained with histogreen and then stained with nuclear fast red. A total of 10 random high-power fields were selected and the number of positive cells was counted.

### Image analysis of HE and Ki-67 staining

An analysis of the necrotic regions was performed using the bio-imaging analysis system (Lumina Vision; Mitani Corporation, Tokyo, Japan). The necrotic regions were assessed based on the inhibition of cytoplasm, denaturation and nuclear fragmentation. In brief, the images of 10 randomly chosen high-power fields (magnification, ×200) in each cross section were captured using a digital camera attached to an Olympus microscope system (Olympus Corporation, Tokyo, Japan). The proportion of the necrotic areas among the total area was calculated. All the tumor tissues were analyzed. The mean proportion of the necrotic areas was calculated.

With regard to the Ki-67 staining, a quantitative digital morphometric analysis of the Ki-67-positive areas was performed. In brief, the images of 10 randomly chosen high-power fields (magnification, ×200) in each cross section were captured using a digital camera attached to an Olympus microscope system (Olympus Corporation). The color wavelengths of the copied image were transformed into digital readings using the Lumina Vision software program (Mitani Corporation), allowing for the quantification of the various color wavelengths, with pixels as the unit of measurement. The proportion of the positive areas in the tumor tissues was calculated by dividing the total pixel area of the positive areas by the total pixel area that corresponded with the total tumor tissue in the field of view. The tumor tissues of three mice in each group were analyzed. The mean proportion of the positive areas in 30 fields was calculated in each group.

### Statistical analysis

The data are expressed as the mean ± SE. The statistical analyses were performed using one-way ANOVA, followed by Tukey-Kramer’s test. P<0.05 was considered to indicate a statistically significant difference.

## Results

### Effects of PHT on tumor growth

**Figure 1:**
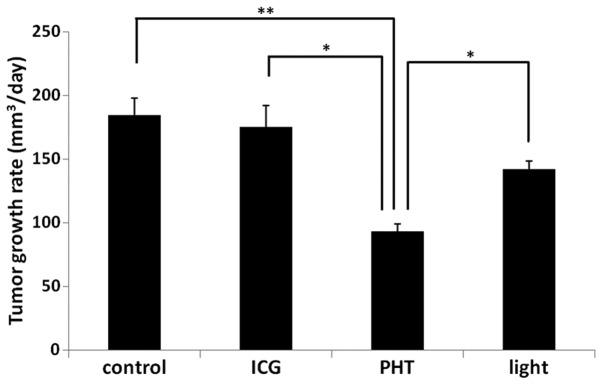
Effects of photodynamic hyperthermal therapy (PHT) on tumor growth. The tumor volume was measured on days 0 and 9. The tumor growth rates (mm^3^/day) were calculated according to the tumor volumes. The data are presented as the mean ± SE of each group. Statistical significance was determined according to the Tukey-Kramer test. ^**^P<0.01 and ^*^P<0.05. ICG, indocyanine green.

The tumor growth rates are shown in Fig. 1. In the PHT group (93.6±5.7 mm^3^/day), the growth rates of the tumor tissues were significantly decreased compared with those observed in the ICG (175.4±16.5 mm^3^/day), light (142.0±6.3 mm^3^/day) and control (184.8±13.0 mm^3^/day) groups (P<0.05).

### Histological observations

In the PHT group, uniform, large necrotic regions were observed at the tumor margin on the skin side. By contrast, numerous foci of necrosis were observed in the light group, primarily at the tumor margin on the skin side. Among the necrotic areas, no immunocytes, including lymphocytes or neutrophils, were observed in any of the groups.

**Figure 2:**
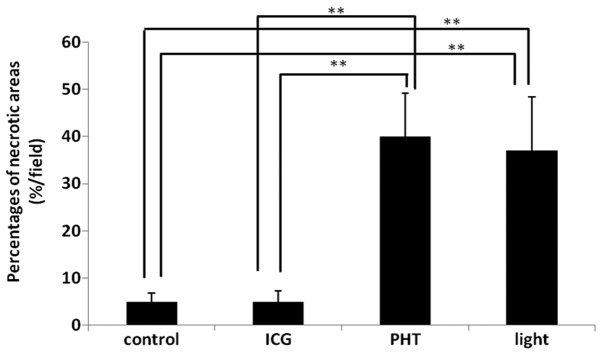
Effects of photodynamic hyperthermal therapy (PHT) on the proportion of necrotic areas in the tumor tissue. The proportions of necrotic areas were calculated. The data are presented as the mean ± SE of each group. Statistical significance was determined according to the Tukey-Kramer test. ^**^P<0.01. ICG, indocyanine green.

The proportions of the necrotic areas are shown in Fig. 2. The values in the PHT (40.0±9.1%) and light (37.0±11.4%) groups were increased significantly compared with those observed in the ICG (5.0±2.3%) and control (5.0±1.8%) groups. However, there were no significant differences between the PHT and light groups.

### Immunohistological analysis

**Figure 3:**
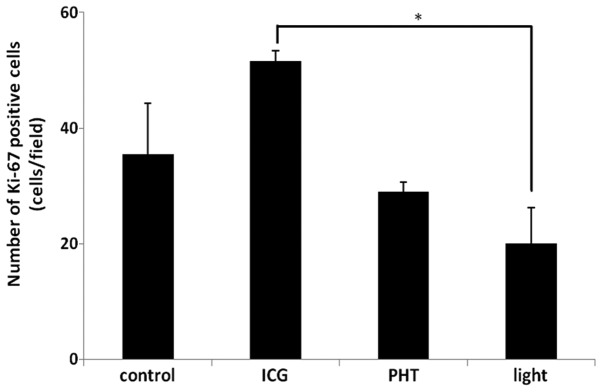
Effects of photodynamic hyperthermal therapy (PHT) on the proportion of Ki-67-positive areas in the tumor tissue. The proportions of Ki-6-positive areas were calculated. The data are presented as the mean ± SE of each group. Statistical significance was determined according to the Tukey-Kramer test. ^*^P<0.05. ICG, indocyanine green.

The results of the Ki-67 immunohistochemistry are shown in Fig. 3. The proportions of the Ki-67-positive areas in the PHT (29.0±1.6%^/^field) and light (20.1±6.1%/field) groups were less than those observed in the ICG (51.6±1.7%/field) and control (35.4±8.9%^/^field) groups. In the light group, the proportion of the Ki-67-positive areas was significantly decreased compared with that observed in the ICG group (P<0.05).

**Figure 4:**
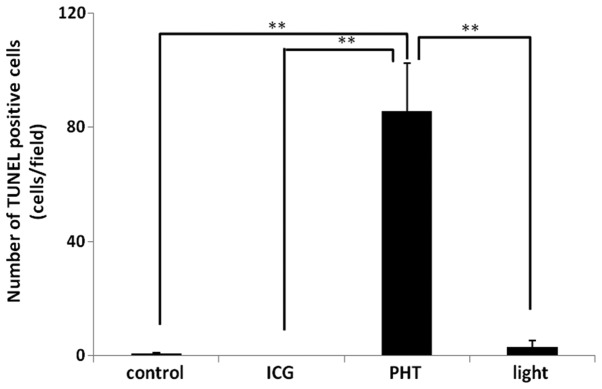
Effects of photodynamic hyperthermal therapy (PHT) on the number of terminal deoxynucleotidyltransferase-mediated dUTP nick end labeling (TUNEL)-positive cells in the tumor tissue. The numbers of TUNEL-positive cells were calculated. The data are presented as the mean ± SE of each group. Statistical significance was determined according to the Tukey-Kramer test. ^**^P<0.01 ICG, indocyanine green.

The results of the TUNEL immunohistochemistry are shown in Fig. 4. The number of TUNEL-positive cells (85.5±16.9 cells/field) in the PHT group was increased significantly compared with that observed in the other groups. In the ICG and control groups, almost no TUNEL-positive cells were observed in the tumor tissues. In the light group, TUNEL-positive cells were observed in the tumor tissues (3.0±2.3 cells/field).

## Discussion

In the present study, PHT was observed to be effective *in vivo* and *in vitro*. The tumor growth rate in the PHT group was decreased significantly compared with that observed in the other groups. It has been reported that the combination of ICG and a 805-nm diode laser exhibits anticancer efficacy *in vivo* and *in vitro* (1–3,12). In these studies, it was speculated that ICG generated heat in response to light near the 800-nm wavelength, which indicated hyperthermia. In the present study, a broadband light source was used instead of a diode laser in the PHT group, as ICG generates active oxygen in response to light at 600–800 nm (photodynamic effect) in addition to hyperthermia (5–8). The present results revealed that PHT is more effective in suppressing tumor growth compared with hyperthermia alone.

Histologically, the proportion of the necrotic areas was similar between the PHT and light groups. This indicates that hyperthermia alone induces tumor necrosis. With regard to the tumor growth rates, the rate that was observed in the PHT group was significantly decreased compared with the rate that was measured in the light group, indicating that the tumor cells in the areas without necrosis in the PHT group proliferated slowly compared with those in the light group.

The slow proliferation of tumor cells implies that numerous tumor cells do not proliferate due to causes such as cell cycle arrest and apoptosis. The present study investigated cell cycle arrest and apoptosis using immunohistochemical methods. TUNEL staining is one method that is used to detect apoptosis (15,16). The present results revealed that the number of TUNEL-positive cells was greater in the PHT group than in the light group. This result indicates that PHT strongly induces apoptosis compared with hyperthermia. Ki-67 is a cell population marker that is detected during all the active phases of the cell cycle, but is absent in resting cells (17). With regard to Ki-67 staining, the proportions of the Ki-67-positive areas in the PHT and light groups were less than those observed in the ICG and control groups, which indicates that PHT and hyperthermia induce cell cycle arrest. However, there were no significant differences between the PHT and light groups. Our previous data has shown that PHT induces cell cycle arrest at an early time following the treatment (14). The present results do not support our previous data. One reason for this discrepancy may be differences in the sampling time following the treatment. In the present study, the samples were obtained at nine days post-treatment. Periodical sampling (days 1, 3, 5 and 7) following treatment is therefore required in future studies.

Tumor cells are more sensitive to heat under acidic conditions (18). Therefore, saline that was adjusted to pH 5.0 using acetic acid was used as a solvent for ICG. In a preliminary experiment, saline that was adjusted to a pH of 4.0 was observed to induce inflammation in the tissue.

In the present study, single treatments were performed, following which, tumor growth was observed. Consequently, it was identified that the single treatments were not adequate to induce complete tumor remission. In order to achieve complete remission, several rounds of treatment are necessary. Further studies are therefore required.

In conclusion, the growth rates of the tumor tissues were significantly decreased in the PHT group. Necrosis and apoptosis were induced by the PHT treatment. The Ki-67-positive areas were significantly decreased by the PHT treatment. The data indicate the PHT has the potential to be a novel cancer treatment. Further studies using clinical patients are required.
